# Oral Health-Related Quality of Life, Self-Care, and Mental Well-Being in Older Adults: Associations in a Biopsychosocial Framework

**DOI:** 10.7759/cureus.113148

**Published:** 2026-07-22

**Authors:** Christos Tsironis, Elena Dragioti, Stefanos Mantzoukas, Agni Nakou, Eleni Bartzou, Nektaria Zagorianakou, Fotios Tatsis, Mary Gouva

**Affiliations:** 1 Scientific Laboratory of Psychology and Person-Centered Care, Department of Nursing, School of Health Sciences, University of Ioannina, Ioannina, GRC; 2 Research Laboratory of Integrated Health, Care and Well-Being, Department of Nursing, School of Health Sciences, University of Ioannina, Ioannina, GRC

**Keywords:** health behavior, mental well-being, older adults, oral health, psychosocial factors, self-care

## Abstract

Background

Oral health is increasingly recognized as a key determinant of overall well-being in older adults. However, its relationship with mental health remains insufficiently understood, particularly in relation to behavioral processes such as self-care. This study aimed to examine the associations among oral health-related quality of life, self-care, and mental health-related quality of life in older adults.

Methodology

A cross-sectional study was conducted among 681 community-dwelling older adults. Oral health-related quality of life was assessed using the Geriatric Oral Health Assessment Index (GOHAI), self-care using the Multidimensional Self-Care Scale for Older Adults (MSCS-OA), and mental health-related quality of life using the Short Form Health Survey-36 Mental Component Summary (SF-36 MCS). Pearson correlation and regression analyses were performed to examine associations among the study variables, adjusting for selected sociodemographic and health-related factors.

Results

Oral health-related quality of life was positively correlated with self-care (r = 0.25, p < 0.001) and mental health-related quality of life (r = 0.39, p < 0.001), while self-care was also positively correlated with mental health-related quality of life (r = 0.38, p < 0.001). In the adjusted hierarchical regression model, both oral health-related quality of life (β = 0.293, p < 0.001) and self-care (β = 0.238, p < 0.001) were positively associated with mental health-related quality of life. The inclusion of self-care accounted for an additional 4.2% of the variance (ΔR² = 0.042, p < 0.001), increasing the total explained variance to 25.8% (R² = 0.258).

Conclusions

Oral health-related quality of life and self-care were positively associated with mental health-related quality of life in older adults. These findings support a biopsychosocial understanding of health and identify self-care as an important behavioral correlate in later life.

## Introduction

Aging represents a complex and multidimensional process that extends beyond biological decline to encompass profound changes in identity, daily functioning, and the individual’s relationship with the body and the social world. In later life, health is no longer defined solely by the absence of disease, but by the capacity to maintain autonomy, functional ability, and meaningful participation in everyday life [1].

The global demographic shift toward an aging population has intensified the need to move beyond traditional biomedical approaches, which conceptualize health primarily in terms of pathology and physiological function. While this model has contributed substantially to scientific progress, it remains limited in its ability to capture the lived experience of health. In response, the biopsychosocial model, introduced by Engel (1977), redefined health as a dynamic interaction of biological, psychological, and social processes, placing human experience at the center of scientific inquiry [2,3].

Within this framework, oral health emerges as a critical yet often underestimated component of overall well-being in older adults. Historically confined to clinical indicators such as dental caries, periodontal disease, and tooth loss, oral health is now increasingly understood as a multidimensional construct shaped by biological, psychological, and social determinants [4,5]. Recent evidence further emphasizes that oral health should be considered a key component of integrated health systems, given its bidirectional relationship with general and mental well-being outcomes [6].

The mouth is far more than an anatomical structure; it is a central medium through which individuals eat, speak, smile, and communicate, thereby maintaining their presence in the social world. Consequently, oral health impairments extend beyond physical discomfort to influence self-image, autonomy, and social participation. Difficulties in chewing, speaking, or appearance may therefore reduce social engagement and ultimately diminish quality of life [4,7].

The concept of oral health-related quality of life reflects this shift toward a more person-centered understanding of health. Rather than focusing exclusively on clinical findings, oral health-related quality of life captures the subjective impact of oral conditions on daily functioning, emotional well-being, and social life [8,9]. This perspective highlights that oral health is not only what can be measured clinically but also what is experienced and lived by the individual. Recent studies reinforce this perspective by demonstrating that oral health-related quality of life is closely associated with mental health trajectories and changes in depressive symptoms over time [10].

In older adulthood, oral health is closely intertwined with broader processes of functional ability, autonomy, and psychological well-being. Age-related changes, combined with social and structural inequalities, may limit access to oral healthcare and contribute to poorer oral health outcomes [4,11]. At the same time, oral health may influence nutrition, communication, and social participation, positioning it as a key determinant of overall quality of life.

Self-care represents an important health-related behavioral factor within this context. In later life, it encompasses engagement in daily health-related routines and practices that support functional independence and well-being [2]. Reduced self-care may coexist with functional limitations, psychological difficulties, or reduced engagement in everyday health-related activities.

Despite growing recognition of the importance of oral health in aging, the associations among oral health-related quality of life, self-care, and mental well-being remain insufficiently explored. In particular, few studies have examined these constructs within a single biopsychosocial framework [10].

The present study aimed to examine the associations among oral health-related quality of life, self-care, and mental well-being in older adults. Specifically, the study investigated the association between oral health-related quality of life and mental well-being and examined whether self-care was independently associated with both constructs within a biopsychosocial framework. Given the cross-sectional design, the study focused on identifying patterns of association rather than causal or mediating relationships.

Hypotheses

Based on the above framework, four hypotheses were examined. First, oral health-related quality of life was expected to be positively associated with self-care in older adults. Second, oral health-related quality of life was expected to be positively associated with mental well-being. Third, self-care was expected to be positively associated with mental well-being. Finally, self-care was expected to explain additional variance in mental well-being beyond oral health-related quality of life and the selected covariates.

## Materials and methods

Study design and participants

This cross-sectional observational study was conducted in Greece between July 2024 and July 2025 as part of a broader research project examining biopsychosocial determinants of health in older adults. Community-dwelling older adults were recruited using a non-probability convenience sampling approach through community centers for older adults, primary healthcare units, municipal social services, and local community associations. This pragmatic strategy was chosen to facilitate the recruitment of eligible community-dwelling older adults across multiple community and primary healthcare settings during the study period. Approximately 700 individuals were approached for participation. An a priori sample size calculation indicated that a minimum sample of 384 participants was required. Eligibility criteria included (a) age ≥ 60 years, (b) community dwelling, (c) ability to communicate in Greek and complete the questionnaire independently, and (d) provision of informed consent. Individuals with severe cognitive impairment or inability to complete the questionnaire reliably were excluded. Following data screening for completeness and consistency, 681 participants who met the eligibility criteria were retained for the study. Missing data in the adjusted regression analyses were handled using listwise deletion.

Procedure

Data were collected using structured questionnaires administered in person. Participants completed the questionnaires independently. When required, a member of the research team provided limited assistance by reading the items aloud or clarifying their wording, without interpreting the questions, suggesting responses, or completing the questionnaire on the participant’s behalf. Given the variability in educational levels among older adults, particular attention was paid to ensuring that all questionnaire items were understood. Participants were informed about the purpose of the study, the voluntary nature of participation, and the confidentiality of their responses. Written informed consent was obtained from all participants before data collection.

Ethical approval was obtained from the Ethics Committee of the University of Ioannina (approval number: 27934; July 24, 2024). All procedures were conducted in accordance with the Declaration of Helsinki and the General Data Protection Regulation. Participants were assured of anonymity and confidentiality and were informed of their right to withdraw from the study at any time without consequence. No sensitive personal data were collected.

Measures

Mental Health-Related Quality of Life

Mental health-related quality of life was assessed using the Mental Component Summary (MCS) of the Short Form Health Survey-36 (SF-36) [12-14]. The MCS was calculated according to the established SF-36 scoring procedure and represents a composite indicator derived from the mental and physical health domains of the SF-36, with greater weighting placed on the mental health-related dimensions. Higher scores indicate better mental health-related quality of life. The MCS is distinct from the individual SF-36 Mental Health subscale and was the outcome used in all correlation and regression analyses in the present study. The SF-36 and its summary component scores have been extensively validated across different populations and have demonstrated satisfactory psychometric properties.

Oral Health-Related Quality of Life

Oral health-related quality of life was assessed using the Geriatric Oral Health Assessment Index (GOHAI) [15]. The GOHAI is a widely used self-report instrument designed to evaluate oral health-related quality of life in older adults across three domains, namely, physical function (e.g., chewing and speaking), psychosocial function, and pain or discomfort. The scale consists of 12 items assessing functional, psychosocial, and pain-related aspects of oral health. Higher scores indicate better perceived oral health-related quality of life. The GOHAI has demonstrated satisfactory reliability and validity across diverse populations. Responses were rated on a six-point frequency scale ranging from 0 (“never”) to 5 (“always”). Negatively worded items (items 1, 2, 4, 6, and 8-12) were reverse-coded, whereas positively worded items (items 3, 5, and 7) retained their original scoring. Item scores were summed to produce a total score ranging from 0 to 60, with higher scores indicating better oral health-related quality of life. Cronbach’s α for the present sample was 0.732. The present study focused on oral health-related quality of life rather than clinical oral examination because its primary aim was to investigate how older adults perceive the impact of oral health on their daily functioning, well-being, and self-care. Oral health-related quality of life represents the subjective experience of oral health and has been shown to be highly relevant in studies examining psychological and behavioral outcomes. Therefore, the validated GOHAI questionnaire was considered the most appropriate measure for addressing the objectives of the present study.

Self-Care

Self-care was assessed using the Multidimensional Self-Care Scale for Older Adults (MSCS-OA) [16], an instrument developed by the authors and psychometrically validated in a sample of 608 community-dwelling older adults in Greece. The validation study has been accepted for publication and is currently in press. Exploratory factor analysis in one subsample (n = 202) and confirmatory factor analysis in an independent subsample (n = 406) supported a 37-item, seven-factor structure comprising functional independence, daily well-being and positive resources, psychological distress and symptoms, prevention and healthcare use, oral hygiene, spirituality/religiosity, and meaning and life purpose. The confirmatory model demonstrated good fit (Comparative Fit Index = 0.982, Tucker-Lewis Index = 0.980, root mean square error of approximation = 0.069, and standardized root mean residual = 0.076).

Items are rated on a five-point Likert scale ranging from 1 (“strongly disagree”) to 5 (“strongly agree”). Subscale scores are calculated as the mean of their constituent items. Higher scores on the psychological distress and symptoms subscale indicate greater symptom burden; however, these items are reverse-scored for calculation of the overall self-care score so that higher total scores consistently indicate greater self-care capacity. The total composite is calculated by summing the 37 retained items after the required reverse scoring, yielding a theoretical range of 37 to 185. No factor-derived or post hoc differential weights are applied: individual items contribute equally, and domains contribute to the total in proportion to their number of retained items.

The total score was used as a global summary index of multidimensional self-care rather than as evidence that the MSCS-OA is a strictly unidimensional instrument. Its use was supported by high three-month test-retest reliability in the validation study (r = 0.86, p < 0.001) and acceptable internal consistency in the present sample (Cronbach’s α = 0.779).

Covariates

Sociodemographic and health-related variables were included as covariates based on theoretical and empirical relevance. These included age (continuous variable), gender (male/female), educational level (categorical), living arrangement (living alone vs. with others), and presence of chronic disease (yes/no). All covariates were included in the regression models to control for potential confounding effects.

Statistical analysis

Descriptive statistics, including means, standard deviations, frequencies, and percentages, were calculated to summarize the characteristics of the sample and the study variables. The internal consistency of the study instruments was assessed using Cronbach’s alpha. Pearson correlation coefficients were computed to examine bivariate associations among oral health-related quality of life, self-care, and mental well-being.

A series of regression analyses was conducted to examine the study hypotheses. First, an adjusted linear regression analysis was performed to assess the association between oral health-related quality of life and self-care. Second, hierarchical linear regression analysis was used to examine predictors of mental well-being. In Model 1, oral health-related quality of life was entered as the focal predictor. In Model 2, self-care was added to determine its incremental contribution to mental well-being beyond that of oral health-related quality of life. Age, gender, educational level, living arrangement, and chronic disease status were included as covariates in all regression models.

For readability, the main regression tables present the coefficients for the primary study variables. Complete adjusted models, including coefficient estimates for all covariates, are provided in Appendices 1 and 2.

To examine whether the association between self-care and mental health-related quality of life was influenced by conceptual overlap between the oral hygiene domain of the MSCS-OA and oral health-related quality of life, a sensitivity analysis was conducted. The self-care composite was recalculated after excluding the oral hygiene domain, and the adjusted hierarchical regression analysis was repeated using the modified composite.

Multicollinearity diagnostics were examined and indicated no significant concerns. Unstandardized regression coefficients (B), standard errors (SEs), standardized regression coefficients (β), t values, 95% confidence intervals (CIs), and p-values were reported. Statistical significance was set at a p-value <0.05. Missing data were minimal and were handled using listwise deletion. The assumptions of linear regression, including normality, linearity, homoscedasticity, and independence of residuals, were assessed and considered adequately met. All analyses were conducted using SPSS Statistics, version 28 (IBM Corp., Armonk, NY, USA).

## Results

Sample characteristics

The final sample consisted of 681 community-dwelling older adults. The mean age was 77.49 years (SD = 8.45). Women comprised 62.9% of the sample, whereas men accounted for 36.5%. Most participants were married (59.6%) or widowed (31.6%), and 29.8% lived alone. Primary education was the most frequently reported educational level (44.0%), and 41.8% of participants reported the presence of a chronic disease. The sociodemographic characteristics of the study sample are presented in Table 1.

**Table 1:** Sociodemographic characteristics of the sample.

Variable	n (%)/Mean (SD)
Age (years)	77.49 (8.45)
Gender	
Male	248 (36.5%)
Female	428 (62.9%)
Marital status	
Single	30 (4.4%)
Married	405 (59.6%)
Divorced	28 (4.1%)
Widowed	215 (31.6%)
Educational level	
Illiterate	71 (10.4%)
Primary education	299 (44.0%)
Secondary education	87 (12.8%)
High school	109 (16.0%)
University	114 (16.8%)
Living arrangement	
Living alone	203 (29.8%)
With spouse	357 (52.4%)
With children	56 (8.2%)
Other	65 (9.5%)
Presence of chronic disease	
Yes	284 (41.8%)
No	396 (58.2%)

Descriptive statistics and correlations

Descriptive statistics and Pearson correlation coefficients among the main study variables are presented in Table 2.

**Table 2:** Descriptive statistics and Pearson correlations among study variables. Note: 1 = oral health-related quality of life (GOHAI); 2 = self-care (MSCS-OA); 3 = mental health-related quality of life (SF-36 MCS). Values below the diagonal are Pearson correlation coefficients. ***: p < 0.001. M = mean; SD = standard deviation; GOHAI = Geriatric Oral Health Assessment Index; MSCS-OA = Multidimensional Self-Care Scale for Older Adults; SF-36 MCS = Short Form Health Survey-36 Mental Component Summary

Variable	M	SD	1	2	3
Oral health-related quality of life (GOHAI)	37.48	7.64	—		
Self-care (MSCS-OA)	128.76	15.18	0.25***	—	
Mental health-related quality of life (SF-36 MCS)	239.12	81.11	0.39***	0.38***	—

Pearson correlation analysis showed that oral health-related quality of life was positively correlated with self-care (r = 0.25, p < 0.001) and mental health-related quality of life (r = 0.39, p < 0.001). Self-care was also positively correlated with mental health-related quality of life (r = 0.38, p < 0.001).

Regression analyses

Association Between Oral Health-Related Quality of Life and Self-Care

Adjusted linear regression analysis (Table 3) showed that oral health-related quality of life was positively associated with self-care (B = 0.184, SE = 0.070, β = 0.093, p = 0.009, 95% CI = 0.046, 0.322).

**Table 3:** Adjusted linear regression analysis predicting self-care (N = 673). Note: The model was adjusted for age, gender, educational level, living arrangement, and chronic disease status. Covariate estimates are omitted from the main table to facilitate readability and are presented in Appendix 1. GOHAI = Geriatric Oral Health Assessment Index; B = unstandardized regression coefficient; SE = standard error; β = standardized regression coefficient; CI = confidence interval

Predictor	B	SE	β	P-value	95% CI
Oral health-related quality of life (GOHAI)	0.184	0.070	0.093	0.009	0.046, 0.322

Predictors of Mental Health-Related Quality of Life

An adjusted hierarchical linear regression analysis was conducted to examine the independent contributions of oral health-related quality of life and self-care to mental health-related quality of life (Table 4).

**Table 4:** Adjusted hierarchical linear regression analysis predicting mental health-related quality of life (SF-36 MCS; N = 672). Note: Both models were adjusted for age, gender, educational level, living arrangement, and chronic disease status. Covariate estimates are omitted from the main table to facilitate readability and are presented in Appendix 2. Model 1 included oral health-related quality of life and the prespecified covariates. Model 2 additionally included self-care. ***: p < .001. MSCS-OA = Multidimensional Self-Care Scale for Older Adults; SF-36 MCS = Short Form Health Survey-36 Mental Component Summary; B = unstandardized regression coefficient; SE = standard error; β = standardized regression coefficient; R² = coefficient of determination; ΔR² = change in explained variance

Predictor	Model 1 B(SE)	β	Model 2 B(SE)	β
Oral health-related quality of life (GOHAI)	3.442 (0.381)	0.324***	3.114 (0.375)	0.293***
Self-care (MSCS-OA)	—	—	1.310 (0.214)	0.238***
Model fit				
Model	R²		ΔR²	
Model 1	0.216		-	
Model 2	0.258		0.042***	

In Model 1, oral health-related quality of life was positively associated with mental health-related quality of life (B = 3.442, SE = 0.381, β = 0.324, p < 0.001), with the model accounting for 21.6% of the variance (R² = 0.216). In Model 2, both oral health-related quality of life (B = 3.114, SE = 0.375, β = 0.293, p < 0.001) and self-care (B = 1.310, SE = 0.214, β = 0.238, p < 0.001) were positively associated with mental health-related quality of life. The inclusion of self-care accounted for an additional 4.2% of the variance (ΔR² = 0.042, F change(1, 661) = 37.33, p < 0.001), increasing the total explained variance to 25.8% (R² = 0.258). Complete adjusted models, including estimates for all prespecified covariates, are presented in Appendices 1 and 2.

**Figure 1:**
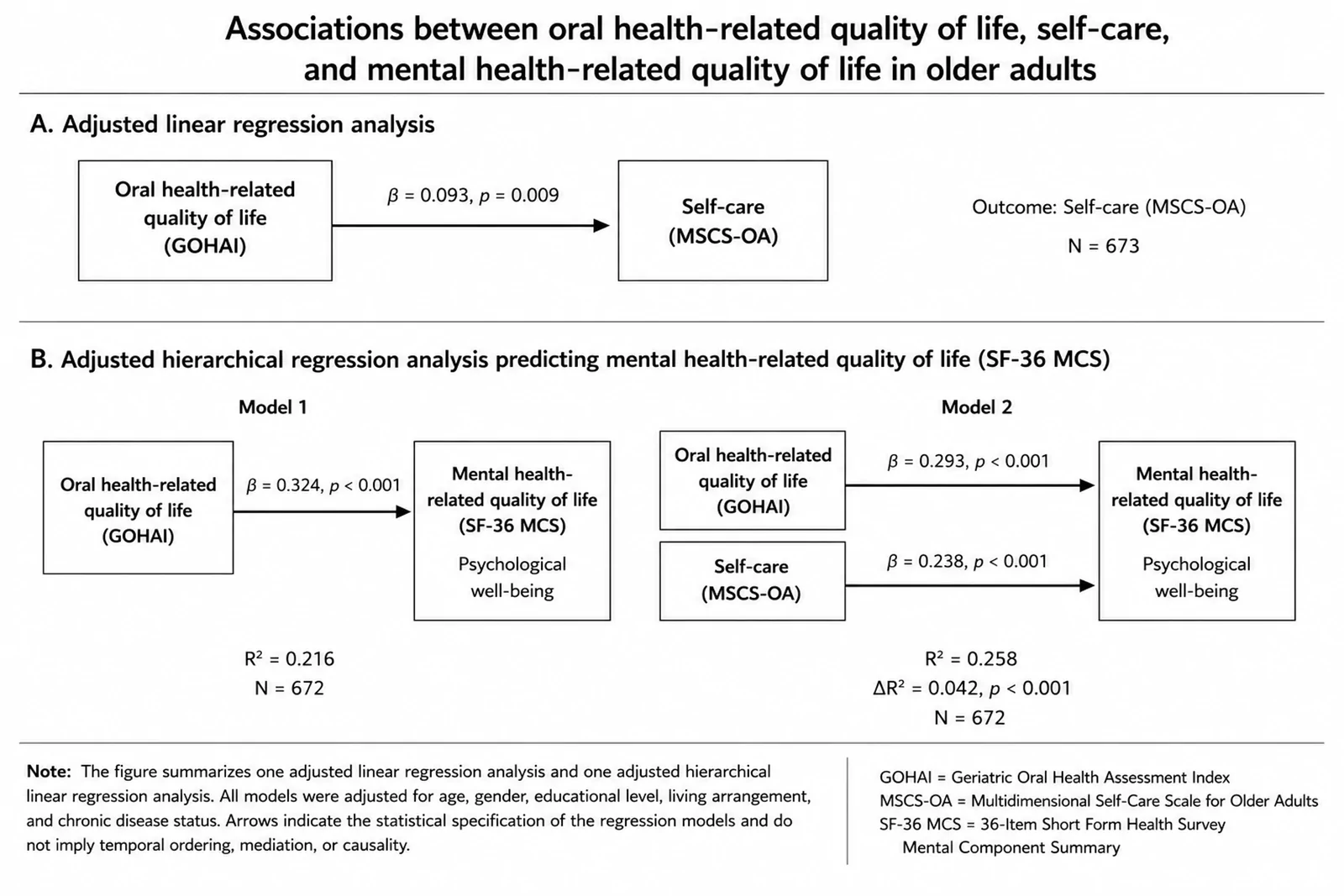
Summary of the adjusted regression analyses examining oral health-related quality of life, self-care, and mental health-related quality of life in older adults. Note: The figure summarizes one adjusted linear regression analysis and one adjusted hierarchical linear regression analysis. All models were adjusted for age, gender, educational level, living arrangement, and chronic disease status. Arrows indicate the statistical specification of the regression models and do not imply temporal ordering, mediation, or causality. GOHAI = Geriatric Oral Health Assessment Index; MSCS-OA = Multidimensional Self-Care Scale for Older Adults; SF-36 MCS = Short Form Health Survey-36 Mental Component Summary

In a sensitivity analysis excluding the oral hygiene domain from the self-care composite, both oral health-related quality of life (B = 3.201, SE = 0.376, β = 0.302, p < 0.001) and the modified self-care score (B = 1.234, SE = 0.230, β = 0.208, p < 0.001) remained positively associated with mental health-related quality of life. The modified self-care score accounted for an additional 3.3% of the variance beyond oral health-related quality of life, and the selected covariates (ΔR² = 0.033), and the final model explained 24.9% of the variance (R² = 0.249). The results of the adjusted regression analyses are summarized in Figure 1.

Taken together, the findings supported the four study hypotheses at the level of statistical association. Oral health-related quality of life was positively correlated with self-care and mental health-related quality of life, and self-care was positively correlated with mental health-related quality of life. In the adjusted hierarchical regression analysis, self-care accounted for additional variance in mental health-related quality of life beyond oral health-related quality of life and the selected covariates. The reduction in the standardized coefficient for oral health-related quality of life after self-care was added reflects a statistical change within the model and should not be interpreted as evidence of mediation or a causal pathway.

## Discussion

The present study examined the associations among oral health-related quality of life, self-care, and mental health-related quality of life in community-dwelling older adults within a biopsychosocial framework. The findings were consistent with the study hypotheses at the level of statistical association. Better oral health-related quality of life was correlated with higher self-care and better mental health-related quality of life, while self-care was also positively correlated with mental health-related quality of life. In the adjusted regression analyses, oral health-related quality of life remained positively associated with both self-care and mental health-related quality of life after accounting for age, gender, educational level, living arrangement, and chronic disease status. Self-care also remained positively associated with mental health-related quality of life and accounted for an additional 4.2% of its variance beyond oral health-related quality of life and the selected covariates. The modest reduction in the oral health-related quality-of-life coefficient after self-care was added represents a change within the statistical model and should not be interpreted as evidence of mediation, temporal ordering, or a causal pathway.

The association between oral health-related quality of life and mental health-related quality of life is consistent with previous research demonstrating that oral conditions are relevant not only to physical functioning but also to emotional well-being and social participation [4,6,7,10]. Difficulties with chewing, speaking, oral discomfort, or dental appearance may interfere with eating, communication, social interaction, and confidence in everyday situations [4,7,17]. These functional and social consequences may help explain why poorer perceived oral health frequently coexists with lower psychological well-being. The present findings therefore support the view that oral health-related quality of life captures aspects of health that may not be identified through clinical indicators alone.

Recent evidence has similarly linked oral health-related quality of life with depressive symptoms and changes in mental well-being over time [6,10]. Nevertheless, the present cross-sectional findings do not establish that poorer oral health precedes or causes poorer mental health-related quality of life. The relationship may also operate in the opposite direction or reflect shared influences. For example, psychological distress, low motivation, limited financial resources, reduced mobility, medication burden, chronic illness, and restricted access to dental services may affect both perceived oral health and mental health-related quality of life. Oral health-related quality of life should therefore be considered as one component of a broader health profile rather than as an isolated determinant of psychological outcomes.

Self-care was also positively associated with mental health-related quality of life after adjustment for the selected covariates. This finding is consistent with research showing that the ability to maintain health-related routines is associated with psychological functioning and well-being in later life [18,19]. In the present study, self-care encompassed functional independence, daily well-being and positive resources, psychological distress and symptoms, prevention and healthcare use, oral hygiene, spirituality/religiosity, and meaning and life purpose. Higher self-care scores may therefore reflect a greater capacity to organize and maintain everyday health-related practices. Conversely, reduced self-care may occur in the context of functional limitations, fatigue, psychological distress, cognitive difficulties, social isolation, or restricted access to supportive services.

The incremental contribution of self-care to the adjusted model was statistically significant but modest. Its inclusion increased the explained variance in mental health-related quality of life from 21.6% to 25.8%. This result indicates that self-care provides information beyond that contributed by oral health-related quality of life and the selected sociodemographic and health-related covariates. It does not, however, demonstrate that self-care mediates or explains the association between oral health-related quality of life and mental health-related quality of life. Formal evaluation of a possible mediating role would be better addressed in longitudinal studies with temporally separated measurements, appropriate control of confounding, and analyses examining whether changes in oral health-related quality of life precede changes in self-care and mental health-related quality of life. Such designs would strengthen the assessment of temporal ordering but would not, by themselves, establish causality.

The present findings can be interpreted within an integrated biopsychosocial framework [2,20], in which oral health, health-related behaviors, psychological functioning, and social circumstances are considered interrelated components of health in later life. Oral health-related quality of life reflects the functional, physical, and psychosocial consequences of oral conditions, while self-care represents the individual’s capacity to maintain daily health-related practices. Mental health-related quality of life reflects broader psychological functioning and well-being.

These components may also be shaped by shared factors such as chronic disease, functional limitations, access to care, social support, socioeconomic conditions, and psychological distress. Oral health should therefore not be viewed solely in clinical terms, but also as an aspect of everyday functioning that may be relevant to eating, communication, social participation, and the maintenance of personal care routines.

In later life, maintaining health may depend not only on the absence of disease but also on the ability to sustain daily routines, obtain appropriate care, and preserve functional independence. Self-care may therefore be considered an important behavioral correlate within the broader association between oral health-related quality of life and mental health-related quality of life. In practical terms, effective care may require attention to clinical oral needs, the ability to perform oral hygiene, access to dental treatment, nutritional consequences, psychological status, and the availability of family or professional support. Integrating these areas may improve the identification of unmet needs and reduce fragmentation between dental, medical, psychological, and social care. However, the present cross-sectional findings do not establish mediation or causal relationships among oral health-related quality of life, self-care, and mental health-related quality of life.

From a dental public health perspective, the findings support closer attention to perceived oral health among older adults. Brief assessment of oral health-related quality of life could be incorporated into primary healthcare, community nursing, geriatric assessment, and municipal services for older adults. Such screening would not replace clinical dental examination, but it may help identify individuals who experience difficulties with chewing, pain, oral discomfort, communication, appearance, or daily oral functioning and who may benefit from referral for dental assessment.

Improving access to geriatric dental care is particularly important for older adults who face mobility restrictions, financial barriers, chronic illness, dependence on caregivers, or limited availability of dental services. Primary care professionals and community health teams could assist with referral pathways to dental services and with the identification of practical barriers that prevent older adults from seeking or completing dental treatment. Greater coordination between dental care, primary care, nursing, and social services may be especially valuable for individuals with multiple chronic conditions or reduced functional independence [4,5,11].

Support for daily oral hygiene should also form part of preventive care. Some older adults may require tailored guidance on toothbrushing, interdental cleaning, dry-mouth management, or the use and maintenance of dental prostheses. Denture assessment and denture hygiene are particularly relevant because poorly fitting or inadequately maintained dentures may contribute to discomfort, eating difficulties, oral infection, and reduced social confidence. Where functional limitations are present, caregivers and family members may need practical education on how to support oral hygiene while preserving the older person’s autonomy and preferences.

The observed associations also suggest that poorer oral health-related quality of life and reduced self-care may warrant broader assessment rather than being viewed solely as dental or behavioral concerns. In primary and community care, persistent oral difficulties, reduced personal care, or limited engagement in established health routines could prompt consideration of functional status, nutritional risk, medication-related oral effects, cognitive difficulties, and psychological distress. Brief mental health screening may be appropriate when markedly poorer oral health-related quality of life or reduced self-care coexists with low mood, reduced motivation, social withdrawal, or diminished daily functioning. Such screening should be understood as an opportunity for further assessment and referral, not as evidence that oral health problems have caused mental illness.

Several limitations should be considered. First, the cross-sectional design does not permit causal inference or determination of the temporal direction of the observed relationships. Second, the study relied on self-report measures, which may be affected by recall error, response tendencies, or social desirability. Oral health was assessed through perceived oral health-related quality of life rather than clinical examination. Consequently, the study could not determine whether the reported difficulties were associated with tooth loss, periodontal disease, caries, oral lesions, dry mouth, denture problems, or other clinical conditions.

Third, convenience sampling may limit the generalizability of the findings to the wider population of older adults. Participants who were able and willing to complete the questionnaires may have differed from individuals with greater frailty, cognitive impairment, institutionalization, or limited engagement with community services. Although severe cognitive impairment was an exclusion criterion, cognitive status was not assessed using a standardized instrument.

Several potentially important confounders were also unavailable, including socioeconomic status or income, dental service use, number of remaining natural teeth, clinical denture status, frailty, previous or current depression, medication burden and polypharmacy, nutritional status, and social support. These factors may influence oral health-related quality of life, self-care, and mental health-related quality of life. Residual confounding therefore remains possible, and the findings should be interpreted as adjusted statistical associations rather than evidence of independent causal effects. In addition, the use of an overall self-care composite may obscure differences among individual domains. Oral hygiene, functional independence, preventive healthcare use, psychological distress, and meaning-related activities may not have equivalent relationships with oral health or mental health-related quality of life.

Future research should use longitudinal and more representative designs to examine the temporal relationships among oral health-related quality of life, self-care, and mental health-related quality of life. Studies should combine validated self-report measures with clinical oral examinations, including assessment of natural teeth, periodontal condition, oral pain, dry mouth, and denture status. Information on dental service use, socioeconomic conditions, medication burden, frailty, cognition, nutrition, depression, and social support would permit more comprehensive adjustment for confounding. Domain-specific analyses of self-care may also clarify whether oral hygiene, functional independence, preventive healthcare engagement, or other components are most closely associated with oral and psychological outcomes. Future analyses examining possible mediation should preferably use longitudinal designs in which oral health-related quality of life, self-care, and mental health-related quality of life are assessed at appropriately separated time points.

## Conclusions

Better oral health-related quality of life and higher self-care were associated with better mental health-related quality of life among community-dwelling older adults. Self-care accounted for additional variance in mental health-related quality of life beyond oral health-related quality of life and the selected sociodemographic and health-related covariates. However, the cross-sectional design does not permit conclusions regarding temporal direction, mediation, or causality. These findings are consistent with a biopsychosocial understanding of health in later life, in which oral functioning, health-related behaviors, psychological functioning, and social circumstances are considered interrelated. They also support closer integration of oral health assessment, self-care support, and broader health evaluation in preventive and community care for older adults. Longitudinal studies incorporating clinical oral examinations are needed to clarify the temporal relationships among these factors.

## Appendices

Appendix 1

**Table 5:** Full adjusted linear regression model predicting self-care (N = 673). Note. Outcome: total MSCS-OA self-care score. The model was adjusted for age, gender, educational level, living arrangement, and chronic disease status. Education was entered categorically. Reference categories were male, illiterate, living alone, and no chronic disease. R² = 0.233; adjusted R² = 0.223; F(9, 663) = 22.41, p < 0.001. Listwise deletion was used. GOHAI = Geriatric Oral Health Assessment Index; MSCS-OA = Multidimensional Self-Care Scale for Older Adults; B = unstandardized regression coefficient; SE = standard error; β = standardized regression coefficient; CI = confidence interval

Predictor	B	SE	β	t	95% CI for B	P-value
Constant	151.504	7.420	—	20.420	136.936, 166.073	<0.001
Oral health-related quality of life (GOHAI)	0.184	0.070	0.093	2.620	0.046, 0.322	0.009
Age (years)	-0.470	0.074	-0.265	-6.324	-0.616, -0.324	<0.001
Female (vs. male)	-1.500	1.589	-0.047	-0.944	-4.619, 1.620	0.346
Primary education (vs. illiterate)	9.134	1.930	0.299	4.733	5.345, 12.924	<0.001
Secondary education (vs. illiterate)	7.083	2.291	0.156	3.092	2.585, 11.581	0.002
High school (vs. illiterate)	11.656	2.301	0.283	5.065	7.137, 16.175	<0.001
University education (vs. illiterate)	11.593	2.277	0.285	5.091	7.121, 16.064	<0.001
Living with others (vs. living alone)	1.009	1.214	0.030	0.831	-1.374, 3.392	0.406
Chronic disease: yes (vs. no)	-4.294	1.598	-0.139	-2.686	-7.432, -1.155	0.007

Appendix 2 

**Table 6:** Full adjusted hierarchical linear regression models predicting mental health-related quality of life (SF-36 MCS; N = 672). Note. Outcome: officially scored SF-36 MCS. Both models were adjusted for age, gender, educational level, living arrangement, and chronic disease status. Education was entered categorically. Reference categories were male, illiterate, living alone, and no chronic disease. Model 1: R² = 0.216, adjusted R² = 0.206, F(9, 662) = 20.32, p < 0.001. Model 2: R² = 0.258, adjusted R² = 0.247, F(10, 661) = 23.02, p < 0.001; ΔR² = 0.042, F change(1, 661) = 37.33, p < 0.001. Listwise deletion was used. GOHAI = Geriatric Oral Health Assessment Index; MSCS-OA = Multidimensional Self-Care Scale for Older Adults; B = unstandardized regression coefficient; SE = standard error; β = standardized regression coefficient; CI = confidence interval

Predictor	B	SE	β	t	95% CI for B	P-value
Model 1: Oral health-related quality of life and covariates						
Constant	211.208	39.936	—	5.289	132.792, 289.625	<0.001
Oral health-related quality of life (GOHAI)	3.442	0.381	0.324	9.030	2.694, 4.191	<0.001
Age (years)	-1.416	0.401	-0.150	-3.532	-2.203, -0.629	<0.001
Female (vs male)	-15.202	8.552	-0.090	-1.778	-31.994, 1.590	0.076
Primary education (vs illiterate)	15.990	10.389	0.098	1.539	-4.409, 36.388	0.124
Secondary education (vs illiterate)	12.410	12.333	0.051	1.006	-11.807, 36.627	0.315
High school (vs illiterate)	26.689	12.397	0.122	2.153	2.348, 51.030	0.032
University education (vs illiterate)	26.416	12.271	0.122	2.153	2.321, 50.511	0.032
Living with others (vs living alone)	10.519	6.532	0.060	1.610	-2.307, 23.346	0.108
Chronic disease: yes (vs no)	-15.022	8.614	-0.092	-1.744	-31.935, 1.892	0.082
Model 2: Oral health-related quality of life, self-care, and covariates						
Constant	12.141	50.731	—	0.239	-87.472, 111.754	0.811
Oral health-related quality of life (GOHAI)	3.114	0.375	0.293	8.304	2.378, 3.851	<0.001
Self-care (MSCS-OA)	1.310	0.214	0.238	6.109	0.889, 1.730	<0.001
Age (years)	-0.756	0.405	-0.080	-1.866	-1.551, 0.039	0.062
Female (vs male)	-13.049	8.334	-0.078	-1.566	-29.413, 3.315	0.118
Primary education (vs illiterate)	4.250	10.296	0.026	0.413	-15.966, 24.467	0.680
Secondary education (vs illiterate)	3.775	12.091	0.016	0.312	-19.966, 27.517	0.755
High school (vs illiterate)	12.370	12.295	0.056	1.006	-11.772, 36.513	0.315
University education (vs illiterate)	12.373	12.167	0.057	1.017	-11.517, 36.263	0.310
Living with others (vs living alone)	9.208	6.364	0.052	1.447	-3.287, 21.704	0.148
Chronic disease: yes (vs no)	-10.249	8.423	-0.062	-1.217	-26.788, 6.290	0.224
